# Time Costs for Genetic Counseling in Preconception Carrier Screening with Genome Sequencing

**DOI:** 10.1007/s10897-017-0205-5

**Published:** 2018-02-08

**Authors:** Frances L. Lynch, Patricia Himes, Marian J. Gilmore, Elissa M. Morris, Jennifer L. Schneider, Tia L. Kauffman, Elizabeth Shuster, Jacob A. Reiss, John F. Dickerson, Michael C. Leo, James V. Davis, Carmit K. McMullen, Benjamin S. Wilfond, Katrina A.B. Goddard

**Affiliations:** 1 Center for Health Research, Kaiser Permanente Northwest, Portland, OR USA; 2 Department of Medical Genetics, Kaiser Permanente Northwest, Portland, OR USA; 3 Seattle Children’s Hospital, Seattle, WA USA

**Keywords:** Whole genome sequencing, Time study, Genetic counseling, Preconception, Carrier testing, Mixed methods

## Abstract

Advances in technology and the promise of personalized health care are driving greater use of genome sequencing (GS) for a variety of clinical scenarios. As health systems consider adopting GS, they need to understand the impact of GS on the organization and cost of care. While research has documented a dramatic decrease in the cost of sequencing and interpreting GS, few studies have examined how GS impacts genetic counseling workloads. This study examined the time needed to provide genetic counseling for GS in the context of preconception carrier screening. Genetic counselors prospectively reported on the time spent in the results disclosure process with 107 study participants who were part of the NextGen study. We found that the median time for results disclosure was 64 min (ranged from 5 to 229 min). Preparation work was the most time-consuming activity. Qualitative data from journal entries, debrief interviews with genetic counselors, and detailed case conference notes provided information on factors influencing time for results disclosure and implications for practice. Results suggest that expanded carrier screening could require significant increases in genetic counseling time, unless we are able to generate new resources to reduce preparation work or develop other strategies such as the creation of new models to deliver this type of service.

## Introduction

Preconception planning is rapidly changing with the introduction of expanded carrier screening (Cho et al. 2013). From a patient and public health perspective, expanded carrier screening can increase the reproductive options for couples planning a pregnancy, allow parents and medical providers to plan for known conditions, and reduce unexpected adverse pregnancy outcomes and infant mortality (Edwards et al. 2015; Grosse et al. 2006). Genome sequencing (GS) has dramatically changed the amount of information that can be provided regarding carrier status, with hundreds of additional possible results to disclose, changing the way in which patients will be counseled (Edwards et al. 2015). Providers of prenatal care have expressed concern that it is critical to have sufficient genetic counseling staff available to ethically provide GS results to patients (Bayefsky et al. 2016), although it is unclear if these same concerns apply in a preconception setting. In addition, there may be alternative approaches to results disclosure, such as disclosure by different types of clinical providers or through online or other methods. But regardless of which type of provider is offering results disclosure, it is important to begin to understand the time needed to provide genetic counseling in the context of large-scale sequencing, and this type of information could be critical to planning for the more widespread incorporation of this technology into clinical practice. Despite this need, there are few empirical estimates of the amount of time that may be needed to provide expanded counseling services associated with large-scale sequencing (McPherson et al. 2008; Bernhardt et al. 1987; Bernhardt and Pyeritz 1989).

As a starting point of understanding the time needed for counseling alongside results disclosure in the context of expanded carrier screening, we conducted a time study as part of the NextGen study, which is investigating the use of GS for preconception carrier testing in a population of women and the reproductive partners of identified carriers (Kauffman et al. 2017). Genetic counselors in the NextGen study conducted results disclosure based upon a list of more than 700 autosomal and X-linked recessive single gene disorders and more than 100 medically actionable incidental findings. The NextGen study provided a unique opportunity to carefully track the time spent by genetic counselors in the entire GS results disclosure process in the preconception context. We sought to determine how much time it takes for genetic counselors to prepare for counseling sessions, disclose GS results, and conduct follow-up activities after results disclosure. We also evaluated which aspects of the genetic counseling results disclosure process are the most time intensive and how the GS results disclosure process differs from current practice in the preconception context.

## Methods

This research was conducted as part of the National Human Genome Research Institute (NHGRI)-funded CSER (Clinical Sequencing Exploratory Research) consortium. CSER focuses on integrating genomic sequencing into clinical care. This paper focuses on a sub-study that explored the time genetic counselors spent on activities related to results disclosure. To answer this question, we used a mixed-methods approach combining quantitative data on time spent in results disclosure activities and qualitative data identifying themes related to results disclosure activities.

### Participants

The NextGen study population included women at Kaiser Permanente Northwest (KPNW) in the Portland, Oregon metropolitan area who had a carrier screening test completed as part of a preconception visit or who had the test during pregnancy and were at least 6 months postpartum, and were planning another pregnancy. All interested women attended an in-person consent visit which included a review of the study protocol and a timeline for how and when results would be delivered, the benefits and limitations of genome sequencing, and a brief genetics educational session. This educational session included a basic overview of genetics, explanation of modes of inheritance for both carrier results and incidental findings, and the potential relevance of results for the patient and their reproductive plans, as well as for family members. If a woman consented to join the study, she was randomly assigned to either genome sequencing (GS) or usual care (UC). Male partners were eligible if their enrolled female partner was found to be a carrier of an autosomal recessive genetic condition through the study-specific genome sequencing. For this sub-study of genetic counselor time use, we only include participants randomized to the GS arm as our focus is on identifying the time necessary for results disclosure in the context of GS. The Kaiser Permanente Northwest Institutional Review Board reviewed and approved all study procedures. All participants provided written consent for participation and received written information on study procedures.

### Genetic Counselors and Clinical Context

The genetic counselors involved in the NextGen study are also clinical genetic counselors within the KPNW-managed care system. These genetic counselors all work in both a medical genetics clinic and perinatology clinic, covering all patient populations, including preconception and prenatal care. At KPNW, routine carrier testing for cystic fibrosis and several other population-specific conditions is most commonly offered, ordered, and resulted by OB/GYN providers (nurses, midwives, doctors); this testing is typically ordered sequentially (not simultaneously for the couple). High-risk results can be routed to the genetic counseling team for further review of results with the patient and other downstream care such as coordination of male partner testing. Increasingly these results are reviewed via phone clinically, although for the research study we returned them in person to allow for additional study activities such as observation of the disclosure visit by qualitative researchers and post-visit debrief sessions with the participant.

### Quantitative Data

We collected time use data using self-report provided by the three study genetic counselors with 6 to 30 years of experience. Genetic counselors were trained in how to categorize and record time using a log, which was completed shortly after task completion.

**Fig. 1:**
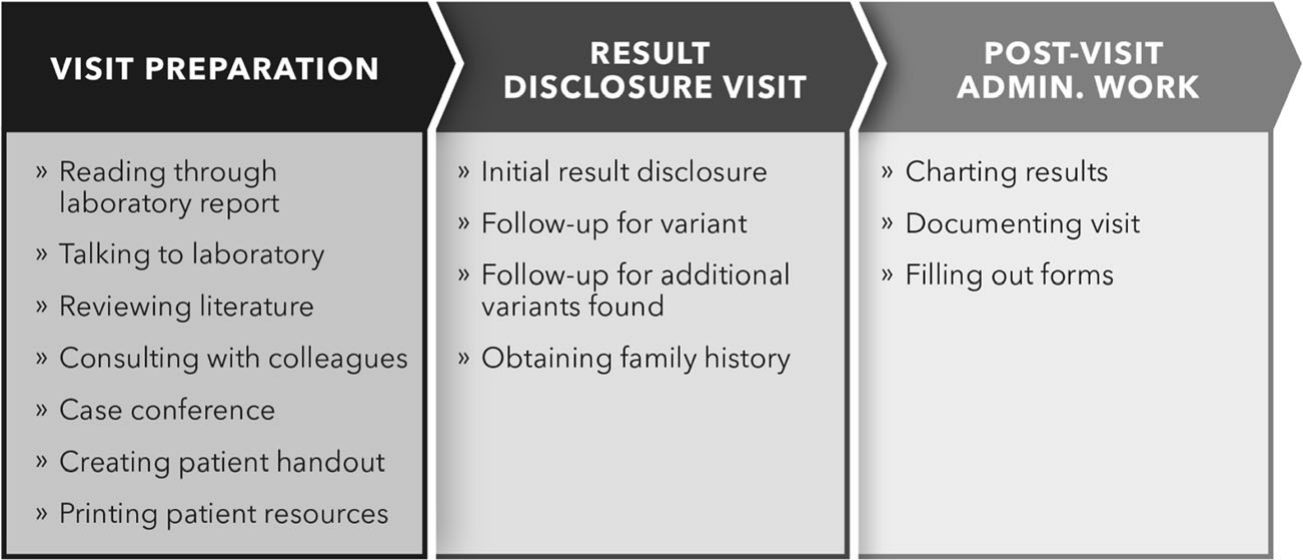
Genetic counseling time categories recorded

Based on pilot results disclosure visits, we developed a list of the specific tasks involved in providing genetic counseling services related to GS for preconception care. We identified three major categories of activities: visit preparation, results disclosure visit with patient, and post-visit administrative work (Fig. 1). Visit preparation included time the genetic counselor spent reviewing lab reports of GS results, communicating with the testing laboratory, researching specific results, communicating with colleagues related to results, and preparing materials for patients. The results disclosure visit included time discussing results with patients and obtaining family histories typically in-person or on the phone. Post-visit activities included charting and documenting results in the EMR, communicating with colleagues, researching any additional questions that came up during the visit, and ensuring that any medically actionable information, based on their results, were communicated to the appropriate clinical team within the healthcare system.

The genetic counselors actively logged data on the time spent in the different study activities and systematically documented the time it took to prepare for the results disclosure visit, counsel study participants, and to provide clinical documentation and follow-up. Two genetic counselors piloted the data collection tool. Questions about how to document and what to document in the different categories were discussed with the NextGen team.

### Quantitative Data Analysis

The data was managed in Microsoft Excel and analyzed in SAS. Analyses included descriptive statistics, such as medians and ranges. Subgroup analyses were conducted related to key factors that were hypothesized to influence time use.

### Qualitative Data

In addition to tracking time spent disclosing results, we analyzed qualitative data pertaining to results disclosure that was collected as part of the NextGen study. We used several types of qualitative data in our analyses. First, study staff (JS, JD, and CKM) who were trained in qualitative methods conducted debrief interviews with genetic counselors after a subset of result disclosure visits. These interviews explored general impressions of the result disclosure process, challenges preparing and communicating results, and potential areas for improvement. Data from debrief interviews were recorded and transcribed. Another source of qualitative data were journal entries (Bunce et al. 2014; Isaacson et al. 2012) written by the genetic counselors that reflected upon their experiences, concerns, and impressions of the GS result disclosure process. Finally, we audio recorded all case conference meetings held among the medical geneticists and genetic counselors as they prepared for and discussed results prior to disclosure. Recordings from these meetings were transcribed into detailed notes.

### Qualitative Data Analysis

Using a thematic content analysis approach (Denzin and Lincoln 2011; Patton 2002; Strauss and Corbin 2008), we reviewed text from 59 case conference session notes, 58 journal entries, and 38 debrief interview transcripts with genetic counselors.

We used a qualitative software program, NVivo (NVivo version 10, 2012), for coding data and creating reports of coded text for analysis. Using the query functions in NVivo, reports were created by searching the text from the three data sources for comments pertaining to “time,” “preparation,” and “research.” These reports were content analyzed and reviewed multiple times. A trained qualitative methodologist (JS) synthesized findings into key themes.

### Description of Clinical Results and Classification of Clinical Encounters

Although a detailed description of the study findings will be reported elsewhere, we include a brief description of the clinical results that were disclosed during the study as part of the characterization of the study population. We included 107 participants in the time study from the 202 total participants who had GS in the NextGen study. All participants in the time study had at least one such result. We identified and disclosed between 1 and 4 carrier results per participant in the time study. Variants were identified in 101 different genes in the time study population, and the most frequently observed genes that were reported in the time study were hemochromatosis (*HFE—*27 carriers), alpha 1-antitrypsin deficiency (*SERPINA1*—10 carriers), *GJB2*-associated deafness (10 carriers), and *ABCA4*-associated Stargardt macular dystrophy/retinal dystrophy (7 carriers). Testing was performed in a CLIA-approved laboratory and we only disclosed pathogenic or likely pathogenic variants to participants.

We defined results as “familiar” if they were common conditions that genetic counselors would routinely encounter in their practice, or if the condition had been previously reported to another study participant in the NextGen study (regardless of whether or not the prior participant was included in the time sub-study population). We classified all other conditions as “unfamiliar.” Thus, the same condition could be classified as “unfamiliar” for the first patient, and “familiar” for the next patient that was encountered who was a carrier for that the same condition. Encounters were also classified according to whether the female partner was pregnant at the time of the encounter. This classification could differ for the male and female partners in a couple since the results were not reported at the same time, and a pregnancy could have occurred in the interim.

## Results

### Participant Characteristics

Participants in the time study (*N* = 107) had similar demographic characteristics to the NextGen study. Participants had a mean age of 33 years, were 78% non-Hispanic white, and tended to have attained a high level of income and education (Table 1). Participants were more likely to be female (74%), reflecting the recruitment strategy where male partners were only invited to join based on the results for the female partner. The female partner was pregnant at the time of the disclosure visit for 18% of the visits. By definition of the inclusion criteria, 100% of the study participants in the time study were a carrier of at least one condition. Of the results disclosed, 42% of participants had familiar results only, 36% of participants had both familiar and unfamiliar results, and 22% of participants had only unfamiliar results.

**Table 1:** Descriptive statistics of sociodemographic characteristics

Characteristic	Total	Male	Female
Mean age, years (SD)	32.9 (4.9)	34.1 (4.8)	32.4 (4.8)
Race/ethnicity			
Non-Hispanic white, *n*	83	24	59
Non-white/multiple, *n*	24	4	20
Education			
Less than Bachelor’s degree, *n*	32	10	22
Bachelor’s degree, *n*	40	12	28
Graduate degree, *n*	34	5	29
Mean number of results per person, *M* (SD)	1.9 (0.9)	1.8 (0.7)	1.9 (0.9)
Pregnant at result disclosure visit, *n*	19	9	10

### Quantitative Results

Table 2 presents the time per participant that genetic counselors spent on results disclosure activities, including time spent preparing for the results disclosure, time spent with the participant during the results disclosure visit, and time spent in clinical and administrative follow-up. Including all 107 participants in the time study, the median total time spent per participant in results disclosure activities was 64 min, with a minimum of 5 min and a maximum time of 229 min. Five participants did not follow through with a genetic counseling visit to receive their results. With these five participants excluded, the median total time was still 64 min (ranged from 22 to 229 min). Preparation time had the most variation per participant, with a median time of 35 min (ranged from 5 to 209 min). Results disclosure and clinical follow-up had narrower ranges of time spent per participant with median times of 30 min for the results disclosure visit (ranged from 0 to 67) and 20 min for clinical follow-up (ranged from 10 to 45) among the 46% with any follow-up recorded.

**Table 2:** Time for results disclosure activities for preconception genomic screening

Time category	Median time (minutes)	Minimum-maximum
All study participants (*N* = 107)		
Preparation time	35	(5–209)
Results disclosure visit	30	(0–67)
Clinical and administrative follow-up	0	(0–45)
Total time in results disclosure process	64	(5–229)
Subgroups of Study Participants		
Pregnancy status		
Pregnant at disclosure (*N* = 19)		
Preparation time	54	(11–209)
Results disclosure visit	30	(0–47)
Clinical and administrative follow-up	0	(15–45)
Total time in results disclosure process	75	(29–229)
Not pregnant at disclosure (*N* = 88)		
Preparation time	34	(5–119)
Results disclosure visit	30	(0–67)
Clinical and administrative follow-up	0	(0–45)
Total time in results disclosure process	63	(5–179)
Number of results disclosed		
1 result only (*N* = 44)		
Preparation time	43	(5–87)
Results disclosure visit	28	(0–60)
Clinical and administrative follow-up	0	(0–45)
Total time in results disclosure process	52	(5–134)
2 results (*N* = 39)		
Preparation time	45	(12–180)
Results disclosure visit	30	(0–60)
Clinical and administrative follow-up	0	(0–45)
Total time in results disclosure process	62	(34–215)
3 or more results (*N* = 24)		
Preparation time	62	(22–209)
Results disclosure visit	30	(0–67)
Clinical and administrative follow-up	15	(0–35)
Total time in results disclosure process	89	(45–229)
Familiar vs unfamiliar result		
Familiar results only (*N* = 45)		
Preparation time	20	(5–70)
Results disclosure visit	30	(0–50)
Clinical and administrative follow-up	0	(0–35)
Total time in results disclosure process	50	(5–111)
Unfamiliar plus familiar (*N* = 38)		
Preparation time	55	(23–94)
Results disclosure visit	30	(0–67)
Clinical and administrative follow-up	15	(0–35)
Total time in results disclosure process	81	(34–161)
Unfamiliar only (*N* = 24)		
Preparation time	53	(24–209)
Results disclosure visit	31	(0–60)
Clinical and administrative follow-up	0	(0–45)
Total time in results disclosure process	84	(41–229)

Table 2 presents time study results for several key subgroups of participants who we anticipated might take more or less time in the results disclosure process. Participants who were currently pregnant at the time of the results disclosure process had a longer total time for results disclosure than those who were not pregnant (median time 75 vs 63 min). This difference resulted primarily from increased preparation time for the visit, which included reviewing the medical record in more detail (e.g., determining gestational age, any genetics screening/aneuploidy results to date, ultrasound results, and whether partner was a KPNW member or not) and determining whether clinical testing of the partner would be available for the gene(s) that were disclosed.

We also looked at how time varied by the number of results that the genetic counselor needed to disclose to the participant. To do this, we divided visits into one result only, two results, or three or more results. We found that median total time spent in the results disclosure process increased as the number of results to disclose increased, with a median of 52 min for one result only, 62 min for two results, and 89 min for three or more results. Again, the main difference was time involved in preparation.

Finally, we looked at the difference in median time for participants that only had familiar results compared to those with one or more unfamiliar results in addition to a familiar result, and those with an unfamiliar result only. We found that those with familiar results only had a lower median total time of 50 min compared to 81 min for those with both familiar and unfamiliar results, and 84 min for those with unfamiliar results only.

### Qualitative Results

To supplement these quantitative estimates, we also conducted a qualitative analysis to provide context as to why time may differ in GS compared to traditional genetic counseling care. This analysis identified factors that influenced time spent in different results disclosure activities. We identified themes regarding factors that increase the time needed for results disclosure, and several factors that might decrease time needed. These results are summarized in Table 3.

**Table 3:** Factors influencing time for results disclosure process

Factor	Illustrative quote
Encountering a very rare genotype	Some of these conditions are so rare, that it’s hard to find information about them. I think I spent 3 or 4 h yesterday just reviewing literature and trying to make sure I had accurate information about the natural history of the condition*.*
Genetic counselor unfamiliar with finding	We both felt like “What is that gene… we did not even know what it was when we started prepping and talking about it!” When it is something we know at least a little bit about, at least we can speak about it in a way where we say “I have talked about this with people before, so I can do this.” But this was like, “Oh I really do not know what this is – I really need to learn about it before I can teach it to someone.”
Preparing for disclosing multiple results	I was surprised by how challenging it is to juggle 5 results that need to be returned. I anticipated that it would be and did my best to prepare, but I do not think anything can prepare you fully for something you have never done before… [genetic counselor reflection on one of the first results disclosed in the study]
Ambiguity in classification of gene	We have identified mutations in 3 patients who have mutations in [the same gene]. Two of them have the same mutation. In one case it was classified as likely pathogenic, in the other case it was classified as pathogenic - so a little bit of inconsistency…I emailed the lab. So either that variant is not as rare as we think it is, or maybe those people are related.
Changes in clinical practice over time	The other interesting thing I learned today, is SLC3A1 associated with cystinuria at the point we returned it, there was no clinical carrier testing in this country. There is now clinical carrier testing…We should be looking into updating, if we get results we have already prepped before.
Need for dedicated time to plan for results disclosure visit	We want to ensure [we have time] to be completely prepared for the visit. This will entail researching the gene/condition association, the range of phenotypes associated with the conditions, the availability of patient-oriented information, determining carrier frequency in the general population and the likelihood of having a child affected with the condition… If there are few reported cases in the literature, the carrier frequency may not be known. In addition, if the condition is rare, there may not be educational materials available for the family. This may present a more difficult counseling situation, and one which is very unusual for us [genetic counselors].

Several factors appear to increase the time needed for results disclosure, including provider uncertainty about the accuracy and interpretation of specific results, and uncertainty about how the results might impact the person receiving the results. Specifically, this is related to variant interpretation in the absence of a phenotype or a family history, especially within the context of genes associated with very rare conditions and where the interpretation may change over time. These factors appear to increase the preparation time needed to provide high-quality results disclosure and highlight the importance of case complexity. It also takes more time to discuss these less certain results with patients. Having multiple results, some being unfamiliar or with uncertainty, and a situation where the patient may not have any prior awareness of a possible concern, makes results disclosure more difficult and time-consuming to explain.

Two themes emerged for factors that reduce genetic counseling time for results disclosure. First, when results are more familiar, or once they have been seen multiple times, preparation time for the results disclosure process decreases. Second, consistent procedures may help to reduce the time in results disclosure. For instance, having the same genetic counselor staff person case manage a patient through the whole results disclosure process may reduce time by eliminating inefficiency from hand-offs among genetic counselors. In addition, the genetic counselors found that use of a structured form for research and case preparation or having regular case conferences to prepare for communication of results helps to streamline the time needed for results disclosure.

We also identified two broad themes related to genetic counselors’ concerns about the expansion of GS into regular clinical practice. The genetic counselors expressed concern about having medical providers who are not specialized in genetics potentially disclosing results to preconception patients. They discussed the possibility that results disclosure might happen in settings outside genetics clinics and by persons not trained in GS results disclosure, such as OB/GYN providers. In current practice, OB/GYNs often disclose preconception and prenatal carrier test results, but expansion of GS results disclosure in these settings might be problematic, because these providers may not have sufficient time available nor expertise to prepare for GS visits.

The idea of incomplete penetrance is really challenging for a lot of people. I wouldn’t want [a patient] to leave thinking his baby had a 25% chance of developing liver disease.

Another genetic counselor further discussed the specific concern that non-genetic specialists would not have the time to adequately research and synthesize more unusual results.

So what is really scalable? What would an OBGYN provider do? …[in genetics] we would have the time in clinical practice to learn about it because we have time in our schedule for prep because we know that is required.

Another difference that genetic counselors noted regarding providing genetic counseling in the context of GS was the overall level of uncertainty regarding results and how to communicate this adequately to patients. This is especially true when there is some ambiguity in the classification of a variant, a lower penetrance condition, or variable expression

I also want to help prepare for the future clinical care downstream. I think to myself, “If I get handed this in the clinical side, what would I do with this?” Also, what if the patient is pregnant, or if she’s pregnant and husband doesn’t want to consent– ‘how’ do I manage that? On the research side, we have gotten comfortable with uncertainty on this, but on the clinical side, I would struggle with what to do.

Finally, the genetic counselors expressed concern about being able to adequately prepare for and communicate GS results in a way that was consistent with the ethical standards of their profession.

It’s a good reminder that you can’t always be working in an ideal situation (enough time to prep), but figuring out that bottom line of ‘what would be ideal and what is still acceptable to provide services,’ – it is a balance of your own [high] expectations versus what is truly needed to provide care for someone.

## Discussion

We described the time required for genetic counseling services for GS in the context of preconception carrier testing and showed that it can be a substantial time commitment, particularly for visit preparation. Factors that increased the time needed for genetic counseling were the participant (or partner) was pregnant at the time of the visit, an increased number of results, and lack of familiarity by the genetic counselors with some of the results. Several previous studies have looked at aspects of time required for genetics services related to GS in other contexts (Arora et al. 2016; Sukenik-Halevy et al. 2016; Williams et al. 2014). However, these studies are substantially different in design and context such that none of these previous reports are easily comparable to the work reported here so direct comparisons are not possible. Williams et al. (2014) described time for pre-test work ups in a neurodevelopmental clinic, but do not provide data on time for the results disclosure process. The other two studies (Arora et al. 2016; Sukenik-Halevy et al. 2016) report on retrospective surveys and provide estimates for genetics practice in general rather than specific contexts, such as preconception care. In addition, one of these studies does not clearly break out estimates of genetic counseling time from that of other genetics providers (Arora et al. 2016).

It is also interesting to view our results in relation to current clinical genetic counseling practice. The 2016 report from the National Society of Genetic Counselors on Work Environments in Genetic Counseling (National Society of Genetic Counselors (NSGC) 2016), for example, provides estimates of time spent in various activities for currently practicing genetic counselors. Results from a survey that included over 2000 practicing genetic counselors found that 72% of respondents spend less than 30 min on preparation for disclosing results, 50% spent less than 30 min face-to-face with patient, and 60% less than 30 min on follow-up. The results of our study suggest that times for preconception results disclosure in the context of GS are somewhat longer, with over 50% of cases taking more than 30 min for both preparation and results disclosure visit times, and over 40% on the higher end of the distribution of total time, with total results disclosure times over 90 min. In addition, respondents to the NSGC survey did not separately report on time spent with patients in different subspecialties of genetics as the report considers genetics practice as a whole. This definition would include time with patients who were undergoing diagnosis of genetic conditions such as cystic fibrosis as well as persons receiving preconception carrier testing. It is also important to note that the NSGC survey required self-report of genetic counseling practice in general (e.g., for the “typical” case) compared to our prospective collection of data on specific cases in a specific context.

Our work adds to the existing research by highlighting the importance of examining preparation time when multiple results are discussed. This may be one of the main areas that is meaningfully different for GS compared to typical clinical genetic practice. In addition, our work suggests the importance of looking at specific contexts (e.g., cancer, pediatrics/adult, preconception/prenatal) because time for genetic counseling services likely differs with different medical contexts. Our study has the unique advantage of investigating the time costs in a healthy population and the inherent uncertainty of interpreting GS results without a disease phenotype.

### Limitations

Although we conducted careful data collection, used prospective methods, and focused on a specific context—preconception screening—there are several limitations that need to be addressed. The generalizability of our findings are limited because we had a relatively small study sample, collected data in only one geographic area, and the patients were primarily well-educated Caucasian adults. Also, results were only disclosed by a small number (*N* = 3) of genetic counselors, and we did not include clinical providers other than genetic counselors, such as medical geneticists or OB/GYN providers. In particular, many OB providers coordinate care for patients who seek expanded carrier testing and deliver these carrier test results, and the time they spend on results disclosure may be different. The current study only included genetic counselors so does not provide time estimates for other types of clinical providers. Future studies exploring both the time needed for other clinical providers (such as OBs) to provide results disclosure and the outcomes of these disclosures could aid significantly in understanding the resources needed to provide results disclosure in the context of expanded carrier testing.

The research study limited the extent of counseling issues addressed to the results themselves and the targeted family history that was obtained (downstream care was referred to the clinical genetics department). Additional common preconception/prenatal counseling issues, such as advanced maternal age, specific family history concerns, or potential teratogen exposure, were not addressed during the course of the results disclosure visit. Thus, the real-world genetic counselor session for GS results disclosure would likely be more time-consuming. We also may have underestimated the time costs because we used a guided list of conditions (Himes et al. 2017) that involved gene-condition associations that were clearly established prior to testing and counseling. Variants of uncertain significance were not reported per study protocol, dramatically reducing the number of results disclosed. Our participants were able to select from five general categories of carrier results, yielding a smaller number of interrogated genes for some participants.

We used self-report of time by the individuals conducting the work, which may lead to bias if reporters are not adequately trained, or if they are motivated to misrepresent data in a way that is desirable for clinic managers or leaders. We have mitigated these risks by providing training and using a log to record times as soon as possible after activities occurred. While alternative approaches include using external observers or computerized systems using time stamps to collect data (Lopetegui et al. 2014), external observers are expensive and would likely have required activity sampling that may not be representative of the distribution of activities under study. Similarly, computerized systems are more useful for medical services where machines or technology are integral, such as dialysis, but less accurate for activities that primarily involve human communication such as genetic counseling. Despite these limitations, this research points to some important considerations for the adoption of GS or even greatly expanded carrier screening into mainstream genetics practice and for future research studies.

### Practice Implications

Although preconception carrier testing by GS is not currently clinically available in some communities, expanded carrier testing panels have been increasing in size. The possibility of incorporating secondary findings into preconception carrier testing will likely increase demands on the clinical providers providing results disclosure. Thus, our results may help health systems plan how to provide genetic counseling services as GS becomes part of standard practice. Our study’s results indicate that if GS becomes the standard of care, many more patients will need preconception genetic counseling services and providers will need to allocate more time to deliver these services. While current professional guidelines recommend preconception carrier screening related to only very few conditions (e.g., cystic fibrosis), the results of our study indicate that many more individuals will have a preconception result with GS, which will also require increased genetic counseling services to help interpret the meaning of that result for their reproductive planning. We found that 78% of study participants are carriers of at least one condition. Thus, even if the number of patients who want preconception carrier testing remains the same (e.g., the number of tests ordered does not increase), expansion of GS to the standard of care would likely substantially increase genetic counseling time. Given that the current genetic counseling work force is relatively small, the growing demand for genetic counseling in laboratory settings as well as clinical settings suggests that we will need substantially more genetic counseling providers if GS becomes the standard of care. This will be critical to address prior to implementation given the time it takes for a person to be trained as a genetic counseling is substantial (Bureau of Labor Statistics (BLS)), and thus, there likely may be a shortage of genetic counseling in clinical settings.

Given the likely shortage of genetic counseling services as use of GS expands throughout medical care contexts, experts have suggested alternative methods for providing genetic counseling in the context of GS. For example, OB/GYN providers often provide genetics results for carrier status for commonly tested conditions such as cystic fibrosis, and some experts have discussed the possibility of such providers disclosing results for GS (Rothwell et al. 2016). A recent ACOG committee opinion on expanded carrier testing recommended referring patients to genetic counseling only when both partners are carrier of the same condition (ACOG 2017). Although this strategy would reduce the genetic counselors’ time demands, the implications for the primary OB providers could potentially be significant as they would be tasked in learning about potentially thousands of conditions and addressing issues about the possibility for the change in interpretation of results over time. In a recent survey of OB/GYNs (Bayefsky et al. 2016), respondents expressed both practical and ethical concerns regarding prenatal GS, but it is unclear if these same concerns would apply to a preconception setting. Results of our qualitative data echo these concerns. Genetic counselors discussed the complex process of preparing for GS results disclosure, describing the need to do extensive research on many results, the need to integrate multiple results, and the importance of having time to put all these factors together prior to the results disclosure visit. As GS becomes more familiar to providers, and additional educational material is developed; however, it may become more realistic for medical staff other than genetic counselors to provide GS results.

Another possible approach to reducing the time required to conduct results disclosure is to complete the visits remotely (Cohen et al. 2013; Tabor et al. 2017; Yu et al. 2013). This includes providing traditional genetic counseling over the phone or by video (Cohen et al. 2013), as well as providing disclosure of GS results through a self-guided management approach (Tabor et al. 2017; Yu et al. 2013). Telephone genetic counseling might mitigate some time needed for GS results disclosure as travel and wait times might decrease, but telephonic genetic counseling is still dependent on the time of genetic counselors. In contrast, self-guided methods might decrease the need for genetic counseling time. For example, My46 is a web tool (Tabor et al. 2017) that guides patients through preferences for what results they want disclosed, educational material regarding results, and helping patients to assess the meaning of the results. While self-guided models hold promise for more efficient results disclosure in some contexts, they have yet to be fully tested in different GS contexts or with a wide range of populations. In addition, they may raise some ethical concerns for some types of results. For example, recent research focusing on carrier testing indicates that patients were highly satisfied with genetic counseling, that genetic counseling improved patients’ understanding of preconception results, and that counseling influenced participant’s reproductive decisions (Rothwell et al. 2016). In contrast, such methods may be more convenient for patients and may become more acceptable over time as both providers and patients become more comfortable with GS.

Our results also suggest several other practical ways which could improve genetic counseling for GS results disclosure in the preconception context. Centralized collection of information on less common results and high-quality patient education materials could reduce the research time needed by practicing genetic counselors and other providers. Another option that could reduce genetic counseling time would be simultaneous testing of both reproductive partners. While this parallel testing would likely increase laboratory testing costs, it would likely reduce genetic counseling time if only at-risk couples are referred for genetic counseling. This approach is not recommended in a recent ACOG committee opinion (2017 ibid.). Another alternative is to limit the scope of the carrier testing through GS in order to maximize clinical utility, and reduce patient stress and clinician time (Stevens et al. 2017). Finally, protocols could be developed to allow primary care providers to provide part of the results of GS disclosure process in low-risk situations such that genetic counseling services would only provide care for more complex or high-risk situations. This is done routinely in OB/GYN clinics for results for single gene testing for common conditions such as cystic fibrosis and Tay Sachs. Such protocols for GS results disclosure, however, do not exist at this point and they would need to be developed and tested prior to adoption in clinical genetics care.

### Research Recommendations

Future research assessing the time providers, including non-genetics providers, require to provide genetic counseling in the context of GS could greatly aid in planning for expansion of GS into preconception care and other areas of medical care. Results of our study suggest that it is important to study time use in specific contexts as general studies may mask important differences in time needed for genetic counseling in different GS contexts. In addition, our results suggest that prospectively collecting data on the amount of time dedicated to these efforts will provide much more accurate results. In addition to aiding in planning for expansion of GS, studying time costs could aid in understanding broad workforce and reimbursement issues (e.g., number of genetic counselors needed) or the cost of a genetic counseling visit in the context of GS. Time studies could also identify tasks that have a high time cost that could be targeted for new interventions, such as development of web-based patient educational materials, or new methods for results disclosure. It would also be very valuable to study how patient understanding of results, satisfaction with results disclosure, and downstream actions following disclosure vary depending on who provides results disclosure and how much time is spent on it. Finally, as public and private insurers grapple with whether or not to include GS in insurance coverage, it is critical to look beyond the laboratory costs of providing GS and ensure that estimates account for genetic counseling and other OB providers’ services, potential prenatal and preimplantation testing, and other downstream activities. Developing accurate and fair health care policies will depend on strong estimates of all the costs of providing GS, and time studies could aid in accurate understanding of what is needed for quality genetic counseling services in the context of GS.
